# Hospital Notes

**Published:** 1904-10

**Authors:** 


### HOSPITAL NOTES.

The Texas Sanitarium for the treatment of tuberculosis has been established at Llano, which is located upon desirable soil with excellent drainage and superb climatic advantages. Llano is 100 miles from Austin, on the Houston and Texas Central Railroad, 1,100 feet above the sea and is in every way adapted to the treatment of tuberculosis. The officers of the institution are: president, J. T. Wilson, Sherman; vice-president, J. W. McLaughlin, Galveston; secretary-treasurer, M. M. Smith, Austin.

The Hospital of the Sisters of Mercy is being established at South Buffalo to serve as an accident or emergency hospital for the reception of sick and injured from the populous and growing section near the Lackawanna Steel plant. The mother house of the Sisters of Mercy recently removed from Batavia to Buffalo. This new hospital will serve a good purpose, such an one being much needed in the location where it is becoming established.

